# Editorial: Magnetic resonance imaging applications for the diagnosis of prostate cancer

**DOI:** 10.3389/fonc.2023.1191543

**Published:** 2023-04-04

**Authors:** Felix Preisser, Michele Marchioni

**Affiliations:** ^1^ Martini-Klinik Prostate Cancer Center, University Hospital Hamburg Eppendorf, Hamburg, Germany; ^2^ Department of Medical Oral and Biotechnological Science, “G.d’Annunzio” University of Chieti, Chieti, Italy

**Keywords:** MRI, prostate cancer, radiomics, imaging, diagnosis

Prostate cancer is the most widely diagnosed disease in men worldwide and is currently ranked as one of the top five cancers in both incidence and mortality. The disease represents significant challenges as prostate cancer has a low survival rate and poor prognosis. Therefore, studies are required to identify how imaging techniques, e.g. magnetic resonance imaging (MRI) can be utilized for early detection and to assess the evolution of metastasis during treatment.

The current Research Topic aims to generate a discussion on how imaging aids in the detection of prostate cancer and which impact it can have on survival. It contains four articles, two systematic reviews and two original articles.

The first review by Turpin et al. focuses on imaging in metastatic prostate cancers in general and its problem. It underlines the dilemma we have in daily practice. With Prostate-specific membrane antigen (PSMA)- positron emission tomography (PET) we have a novel imaging modality with better sensitivity and specificity compared to conventional imaging technologies. However, its availability is limited, and outcomes of most randomized trials still rely on conventional imaging. Next to a validation of novel imaging modalities, the authors highlight the importance of a better communication between clinicians and imagers.

The second systematic review and meta-analysis by Xie et al. compares the detection rates of prostate cancer with MRI/Transrectal Ultrasound Fusion-Guided Targeted Biopsy and Transrectal Ultrasound-Guided Systematic Biopsy. 26 studies could be included in the meta-analysis. The results clearly underline how crucial prostate MRI is in the diagnostic workout. Compared to systematic biopsies, MRI-targeted biopsy alone detects more clinically significant and more high-risk prostate cancer cases, and fewer clinically insignificant cases. MRI-targeted biopsy combined with systematic biopsy enhances the overall detection rate compared to either one of the biopsy techniques alone but does not reduce the detection rate of clinically insignificant disease.

The original work by Yang et al. aims to assess the association of radiomics features based on multiparametric MRI (mpMRI) with the proportion of intraductal carcinoma of prostate. Within a cohort of 97 men, the authors could demonstrate how radiomics can help to differentiate between low and high proportions of intraductal carcinoma and pure acinar adenocarcinoma. It gives us a hint what radiomics based on MRI information can do and how it might influence diagnostic and treatment decisions in future.

The last prospective study of this Research Topic by Reijnen et al. explores if a high-resolution diffusion weighted MRI sequence (DWI-only) could be used as a first step in an MRI-directed diagnostic pathway. The authors could demonstrate that with DWI-only, all patients diagnosed with clinically significant cancer were identified. These findings are important. Reducing the multiparametric MRI to an monoparametric, or a biparametric MRI, which is currently assessed in the Prostate Imaging Using MRI +/- Contrast Enhancement (PRIME) trial (NCT04571840), MRI costs as well as exam time could be significantly reduced and thus be available for more patients.

In summary, this Research Topic contains several high-quality articles, reviewing the current literature and raises new concepts and ideas related to the role of MRI and imaging in prostate cancer that can also be used as further insights for work in this field.

## Author contributions

FP wrote the first draft of the manuscript. MM contributed to manuscript revision, read, and approved the submitted version.

